# Cooperation dynamics in dynamical networks with history-based decisions

**DOI:** 10.1371/journal.pone.0275909

**Published:** 2022-11-15

**Authors:** Adam Lee Miles, Matteo Cavaliere

**Affiliations:** Department of Computing & Mathematics, Manchester Metropolitan University, Manchester, Lancashire, United Kingdom; Teesside University, UNITED KINGDOM

## Abstract

In many aspects of life on earth, individuals may engage in cooperation with others to contribute towards a goal they may share, which can also ensure self-preservation. In evolutionary game theory, the act of cooperation can be considered as an altruistic act of an individual producing some form of benefit or commodity that can be utilised by others they are associated with, which comes at some personal cost. Under certain conditions, individuals make use of information that they are able to perceive within a group in order to aid with their choices for who they should associate themselves within these cooperative scenarios. However, cooperative individuals can be taken advantage of by opportunistic defectors, which can cause significant disruption to the population. We study a model where the decision to establish interactions with potential partners is based on the opportune integration of the individual’s private ability to perceive the intentions of others (private information) and the observation of the population, information that is available to every individual (public information). When public information is restricted to a potential partners current connection count, the population becomes highly cooperative but rather unstable with frequent invasions of cheaters and recoveries of cooperation. However, when public information considers the previous decisions of the individuals (accepted / rejected connections) the population is slightly less cooperative but more stable. Generally, we find that allowing the observation of previous decisions, as part of the available public information, can often lead to more stable but fragmented and less prosperous networks. Our results highlight that the ability to observe previous individual decisions, balanced by individuals personal information, represents an important aspect of the interplay between individual decision-making and the resilience of cooperation in structured populations.

## 1 Introduction

In many facets of life on earth, it is possible to observe cooperation between individual entities. This phenomena can be observed within the foundations of modern society including the global economy and within politics across the globe [1–4]. The occurrence of cooperation can also be observed within natural contexts including animal social groups and cellular biology [5, 6]. When groups such as these are modelled, it becomes possible to analyse the finer dynamics that can occur within these groups [7, 8]. The dynamics of these networks can be further expanded upon by considering how interactions between individuals can affect their well-being and the decisions they undertake, this is commonly studied as part of the field of evolutionary game theory [6].

Under game theory, a cooperator can be considered as an individual that will pay some of cost in order for other individuals to receive some form of benefit [2, 9]. For example, a bee, as part of a colony, may put aside it’s own reproductive potential in order to protect the colony, this act will in turn raise the reproductive potential of other bees [6]. In an ideal scenario, other individuals will engage in like-minded behaviour and contribute towards cooperation within these groups, which allows for benefits to be produced for all associated individuals and make up for the personal costs incurred [10]. This should result in a scenario where all individuals are able to look out for each other whilst potentially working towards a shared goal. The scale of these kinds of interactions can range between just two individuals to large groups of interconnected individuals, each with a varying amount of connectivity. This can be used to represent different kinds of communities of varying scale and connectivity.

All individuals, when engaging in cooperative behaviour, can collectively produce benefits for the group and support group well-being. However, as seen in various games [6] and historical events [11], this altruistic behaviour can be taken advantage of by opportunistic defectors that may be present. In game theory, a defector is considered as an individual that engages in actions that improves their own payoff, but results in an overall socially inefficient outcome. However, a defector will still take advantage of the benefits produced by other cooperators it is associated with, giving nothing in return. A game that is commonly used to illustrate this conflict is the prisoner’s dilemma [4]. Here, both players have the choice to either cooperate with or defect against their opponent. When examining the payoff matrix of this game, it becomes clear that the most logical strategy to adopt is defection in order to maximise potential benefits whilst mitigating any losses. The conflict between cooperation and defection that can arise in these scenarios is commonly studied in the field of evolutionary game theory [6, 12], where the fitness of individuals within these scenarios are taken into consideration. Depending on how fitness is defined within an area of study, the fitness of individuals can change over time [13–15] in response to changes in their local environment, can affect how well they perform and can also affect the likelihood of reproducing [16].

This paper expands upon previous work by Yang et al [17]. The previous presented results demonstrate how the presence of public and private information can affect the connections formed by individuals as they join a network. The connections that a newcomer may form are limited to a chosen role-model and to all individuals that are currently associated with the role-model. The choice of role-model selected by an individual will also influence the strategy that will be adopted when joining the network, determining how it will interact with others it forms connections with. The choice of role-model is influenced by individual fitness of individuals present within a network. Individuals make use of two kinds of information, public and private. Private information is based on an individuals ability to perceive if a node is trustworthy and public information is based on the number of connections a node has and if that number exceeds the network average. These two kinds of information are utilised by individuals when joining a network and determining which individuals it should form a connection with.

However, in the model studied in [17], the kind of public information available to newcomers is restricted to observing the current number of accepted connections a potential neighbour possesses. The findings also illustrate how the presence of public information can lead to the emergence of information cascades, which can potentially contribute to the collapse of cooperation. This method of observing the connections an individual possesses limits newcomers to observing an individual at the current moment and does not allow newcomers to also consider historical actions that have been taken by other newcomers. This type of public information also limits newcomers to a simple ‘yes or no’ evaluation as opposed to more nuanced consideration. In previous works [18–20], the past actions of other individuals are considered by others when determining a course of action to take, illustrating that the range of information available may not necessarily be limited to the current moment in time. The information made available to individuals can also have some additional influence on decision-making, which could potentially push them towards making decisions they may have not ordinarily taken [18]. Although, these previous works have not considered the consequences that such types of history-based decision making can have on the ability of a population to cooperate. This paper explores how the presence of public information that is based on the historical decisions of previous newcomers can have an effect on cooperation, prosperity and instability in a structured population.

To evaluate the dynamics of these networks, we make use of computer simulations. Networks in these simulations are subjected to different methods of interpreting public information with a varying amount of information being available to newcomers. We determine how adjusting these factors can affect the level of cooperation in these networks and their underlying topology.

## 2 Materials and methods

### 2.1 Computational model

The model utilised for this paper expands upon previous work by Yang et al [17], in order to explore how the presence of public information as an observable history of previous decisions can affect cooperation and network well-being. In the model utilised by Yang et al [17], newcomers joining a network of a fixed size select a role-model from individuals already present in the network based on their level of fitness. A newcomer will then make use of both public and private information to determine which individuals it should form connections with. Any connected individuals will engage in a round of a donation game.

We utilise a special case of the prisoner’s dilemma game [6] to investigate the conflict that can emerge between cooperators and defectors, called a donation game. Here, an individual may engage in cooperative behaviour to produce benefits for each of its neighbours, which will come at some personal cost. An individual engaging in defection will produce no benefits incurring no costs whilst still taking any benefits produced by others. The payoff matrix utilises parameter *b*, which determines the value of benefits produced from a cooperative individual. Parameter *c* is used to determine the cost that is incurred when an individual engages in cooperation. Throughout simulations, we assume that *b* is set to 10 and *c* is set to 8, which will adhere to the condition of *b* > *c* > 0. The payoff *P*_*i*_ of individuals is determined by the sum of all payoff obtained by all interactions that individuals have with their neighbours. For example, assuming a cooperator has *K* cooperative neighbours and *J* defective neighbours, they will receive the payoff described in Eq (1). For defectors, assuming they have *K* cooperative neighbours and *J* defective neighbours, will instead receive a payoff described in Eq (2).

$$\begin{array}{c} K(b-c)+J(-c) \end{array}$$ (1)

$$\begin{array}{c} K(b)+J(0) \end{array}$$ (2)

Parameter *δ* is used to determine the level of selection strength that is present in networks and will influence the likelihood that nodes are selected as role-models based on their payoff *P*. This parameter is used to calculate the fitness of a node *i* as described in Eq (3).

$$\begin{array}{c} \left(1+\delta \right)P_{i} \end{array}$$ (3)

**Fig 1:**
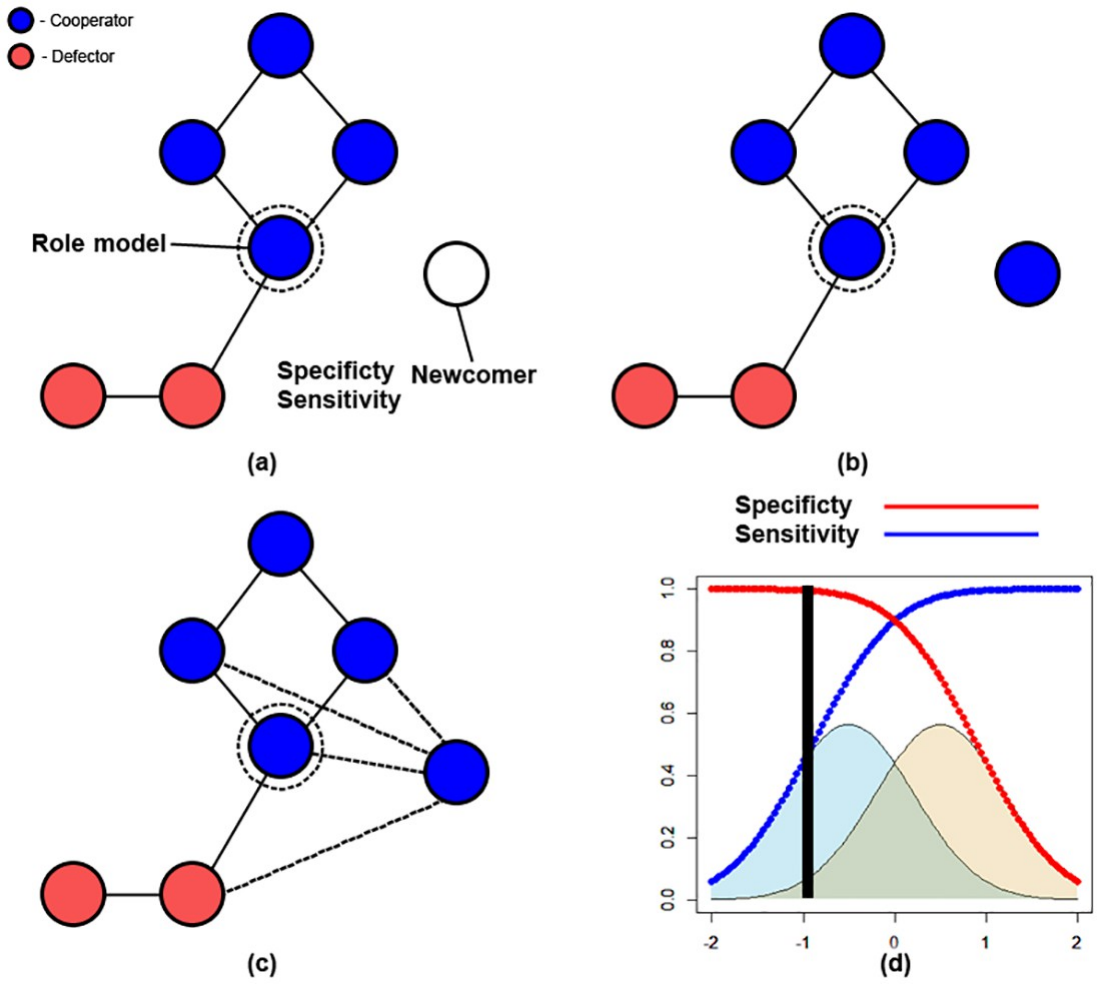
An illustration of the process that is undertaken as a newcomer joins the network at each step during the simulation. Firstly, a newcomer will select an existing node to act as its role-model, which is influenced by node fitness and *δ* = 0.001 (a). Following this, the newcomer will adopt a strategy, by either copying the role-model or mutating by adopting the opposing strategy (b). Lastly, the newcomer will then proceed to choose which of the role-models neighbours to form connections with, which is done by making use of available public and private information (c). To model private information, two Gaussian distributions are used to represent an individuals ability to identify either cooperators or defectors respectively (d). Specificity and sensitivity represents an individuals ability to correctly reject a connection with a defector or correctly accept a connection with a cooperator respectively.

At each step during a simulation, a newcomer is incorporated into the network (Fig 1). Firstly, an existing node is randomly selected to act as the newcomers role-model (Fig 1a). This selection is influenced the fitness of nodes, obtained as in Eq (3), with a higher value meaning a node is more likely to be chosen as a role-model over other nodes. Next, the newcomer will determine if it will adopt the strategy of its role-model (Fig 1b). Parameter *μ* is used to determine the likelihood that a mutation will occur. Following [17], *μ* is set to 0.0001 to allow for comparison against previous work. If a mutation occurs, the newcomer will instead adopt the opposing strategy of its role-model. The role of mutation within networks is to avoid networks only consisting of either cooperators or defectors, and allows for the possibility of a previously absent strategy to invade a network. Following [17], public and private information are then used to establish which connections the newcomer will form. The potential connections an individual may form are restricted to the chosen role-model and any individuals that are connected to the role-model, and is done using the decision-making process described in Section 2.4 (Fig 1c).

### 2.2 Private information

Following [17], we utilise two fixed Gaussian distributions (See Fig 1d) to model a newcomers ability to determine the intentions of a potential neighbour as private information. Both distributions are created with a variance of *σ*^2^ = 0.5 and both peaks are set 1 apart from each other, with a *μ* of -0.5 for *φ*_*c*_ and a *μ* of 0.5 for *φ*_*d*_. The use of these two overlapping distributions *φ*_*c*_ and *φ*_*d*_ are used in order to emulate an individuals imperfect ability to differentiate between potential cooperators and defectors respectively. For each node *x* (either the role-model or any nodes connected to the role model) where a connection can be formed, a value is sampled from either *φ*_*c*_ if the node *x* is a cooperator or *φ*_*d*_ if node *x* is a defector. Should the *decision* *threshold* *τ* exceed the sampled value, the private information will indicate that a connection should be made towards node *x*. Otherwise, the private information will indicate that a connection should not be made towards *x*. Increasing *τ* throughout simulations increases the likelihood that private information will indicate that a connection should be made. Whilst this will mean that newcomers are open to form more connections, this also means that more connections with defectors are increasingly likely (see Fig 1d).

### 2.3 Public information

#### 2.3.1 Connection average

**Fig 2:**
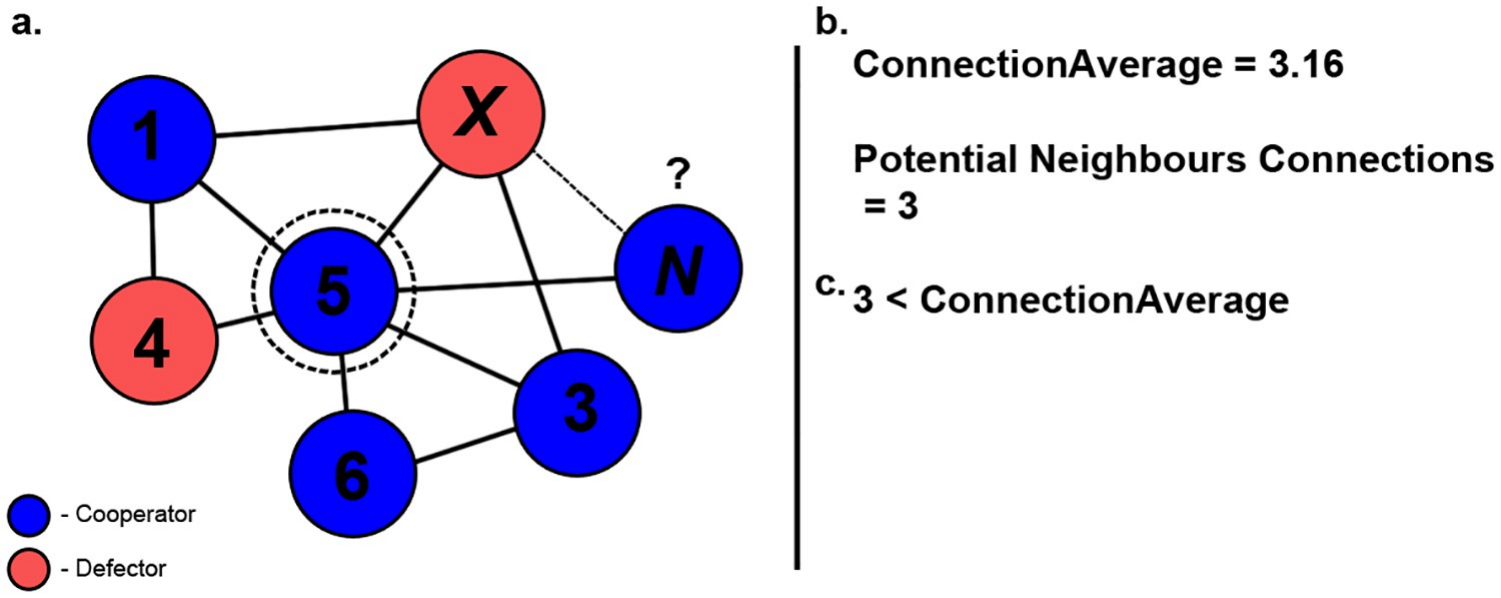
Example scenario of connection average method being utilised. Here, the newcomer is determining if it should form a connection with node x (a). The connection average across the network and the current connection count of x is obtained (b). As the current connection count for node x does not exceed the network average, public information will indicate that a connection should not be made (c).

To understand how public information based on previous decisions can influence structured populations. we review a type of public information, referred to as connection average (see Fig 2), that has been studied in previous works [17, 21], which does not consider previous decisions but only the current connections of a potential neighbour. In this case, public information will be obtained by examining the number of connections a node *x* possesses and if that count exceeds the average connection count across the network.

Given the node *x*, public information will give an indication whether or not a connection should be formed with *x*, based on the following conditional statements:

- If node *x* has connection count that exceeds connection average, public information will indicate that a connection should be formed with *x*
- If node *x* has connection count that does not exceed connection average, public information will indicate that a connection should not be formed with *x*

#### 2.3.2 Global history

**Fig 3:**
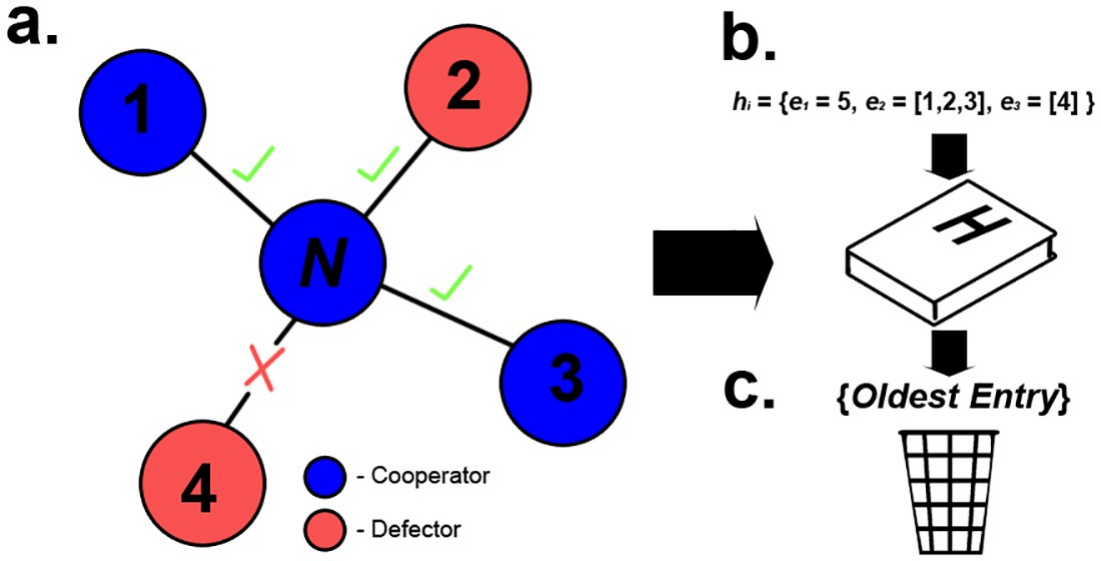
Process of appending new information to the global history list *H*. As newcomers *N* determine which connections they form with other nodes *x*, the connections they accepted and rejected are noted (a). Following a newcomer *N* joining the network, a tuple *h*_*i*_ comprised of the newcomer ID *e*_1_ and all accepted *e*_2_ and rejected *e*_3_ connection decisions are appended into *H* (b). The oldest log is also removed from *H*, keeping *H* from exceeding a size of *S* (c).

We expand upon previous work [17] by considering a kind of public information based on the sequence of decisions undertaken by previous newcomers which we generally refer to as ‘global history’ (as it represents the global history of decisions taken by previous newcomers). Here, we introduce global history into the model by adding to the model a list *H* = {*h*_1_, *h*_2_…*h*_*n*_} of recorded previous undertaken decisions by newcomers (i,e., *H* represents then the ‘global history’). After a newcomer has joined a network, a 3-tuple entry *h*_*i*_ = {*e*_1_, *e*_2_, *e*_3_} is appended to *H*, recording the accepted and rejected connections during decision making (see Fig 3b). For each entry *h*_*i*_, *e*_1_ is used to record the ID of the newcomer that this entry in *H* relates to. *e*_2_ and *e*_3_ records the individuals the newcomer accepted (*a*) and rejected(*r*) connections with respectively. To restrict the amount of information available to newcomers, we implement parameter *S* to define the number of entries that can be present in *H*. When a newcomers actions are logged, and when the length of *H* exceeds *S*, the oldest entry is removed from *H*. To ensure that information present in *H* is accurate and to account for the Moran process, any accepted or rejected information relating to a node *x* is removed from *H* if that node *x* is removed by a newcomer. We define *AcceptTotal*_*x*_ as the total number of accepted connections made with a node *x* present on the network that are currently recorded in *H*. We define *RejectTotal*_*x*_ as the total number of connections that are rejected towards a node *x* present on the network that are currently recorded in *H*. We define *fAcceptIndexes*^*H*^(*x*) as a list of indexes that correspond to the position of entries in *H* where a connection to the specified node *x* was accepted. We define *fRejectIndexes*^*H*^(*x*) as a list of indexes that correspond to the position of entries in *H* where a connection to the specified node *x* was rejected.

**Fig 4:**
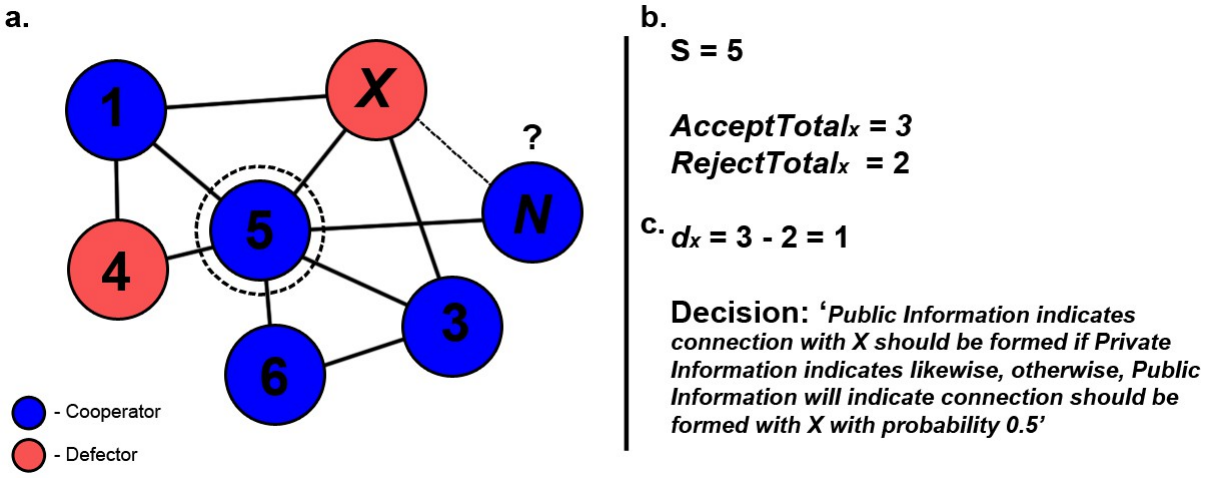
Example scenario of ‘Bikchandani’ method being utilised. Here, the newcomer is determining if it should form a connection with node x (a). The total number of accepted and rejected connections for node x are obtained (b). With these values, the difference *d*_*x*_ is calculated and the final decision of this evaluation is based on the conditional statements that have been defined for this method (c).

The first of the methods using the global history is called ‘Bikchandani Method’, based on previous works carried out by Bikchandani et al [18]. When utilising this method, a newcomer will examine the difference *d*_*x*_ between the total previously accepted *AcceptTotal*_*x*_ and rejected *RejectTotal*_*x*_ connections for node *x*(see Fig 4). For this method, should the difference exceed either 1 or -1, the indication from private information will be overwritten to match the given indication from public information to either accept or reject a connection.

Given the node *x*, public information will give an indication whether or not a connection should be formed with *x*, based on the following conditional statements:

- If *d*_*x*_ > 1, then public information and private information will indicate connection should be formed with node *x*, the previous indication from private information is overwritten
- If *d*_*x*_ = 1, then public information will indicate a connection should be formed with node *x* if private information also indicates likewise, otherwise, public information will indicate connection should be formed with node *x* with probability 0.5.
- If *d*_*x*_ = 0, then public information will indicate connection should not be formed with node *x*
- If *d*_*x*_ = −1, then public information will indicate a connection should not be formed with node *x* if private information also indicates likewise, otherwise, public information will indicate connection should not be formed with node *x* with probability 0.5
- If *d*_*x*_ < −1, then public information and private information will indicate connection should not be formed with node *x*, the previous indication from private information is overwritten

**Fig 5:**
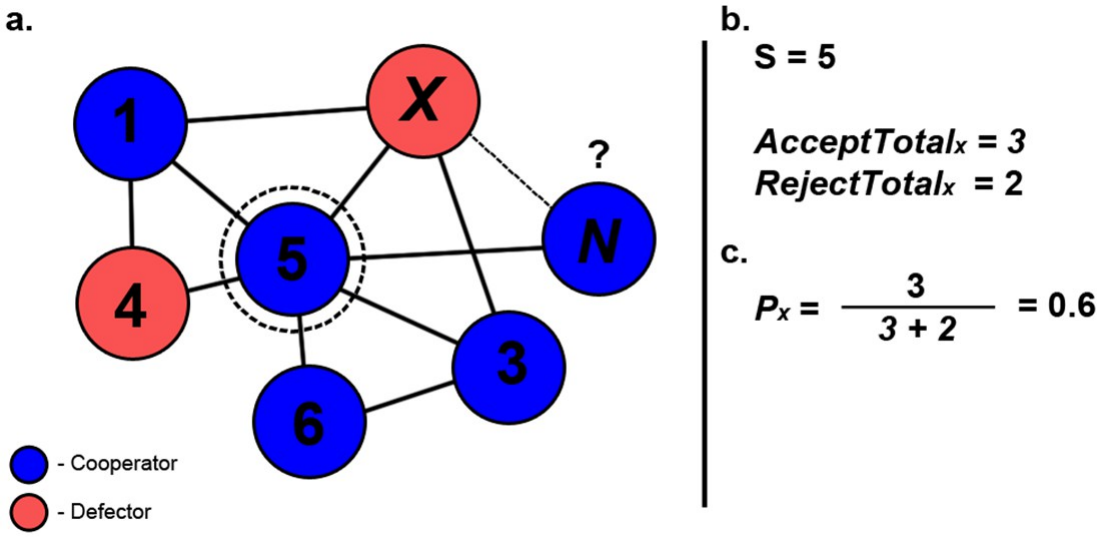
Example scenario of probabilistic aggregation method being utilised. Here, the newcomer is determining if it should form a connection with node x (a). The total number of accepted and rejected connections for node x are obtained (b). These values are then used to calculate *P*_*x*_, which determines the likelihood of public information indicating a connection should be made (c).

The second method to use global history implemented into the model we refer to as ‘Probabilistic Aggregation’. The intuition behind this method is that public information will be more likely to indicate a connection should be formed where a node *x* has more previously accepted connections towards it than rejections (see Fig 5). The probability *P*_*x*_ calculated for this method is described in (4), where *AcceptTotal*_*x*_ and *RejectTotal*_*x*_ are defined in Section 2.4. *P*_*x*_ will determine the likelihood that public information will indicate a connection should be formed based on the information currently stored in *H* for a node *x*. When evaluating a prospective neighbour *x*, if less than two entries of both accepted and rejected connections are available, a newcomer will instead utilise a coin-toss to determine if public information will indicate a connection should be made with node *x*.

Given the node *x*, public information will give an indication whether or not a connection should be formed with *x*, based on the following conditional statements:

- If *AcceptTotal*_*x*_+ *RejectTotal*_*x*_ > 1, then public information will indicate a connection should be formed with node *x* with probability *P*_*x*_
- If *AcceptTotal*_*x*_+ *RejectTotal*_*x*_ ≤ 1, then public information will indicate a connection should be formed with node *x* with probability 0.5

$$\begin{array}{c} P_{x}=\frac{AcceptTotal_{x}}{AcceptTotal_{x}+RejectTotal_{x}} \end{array}$$ (4)

**Fig 6:**
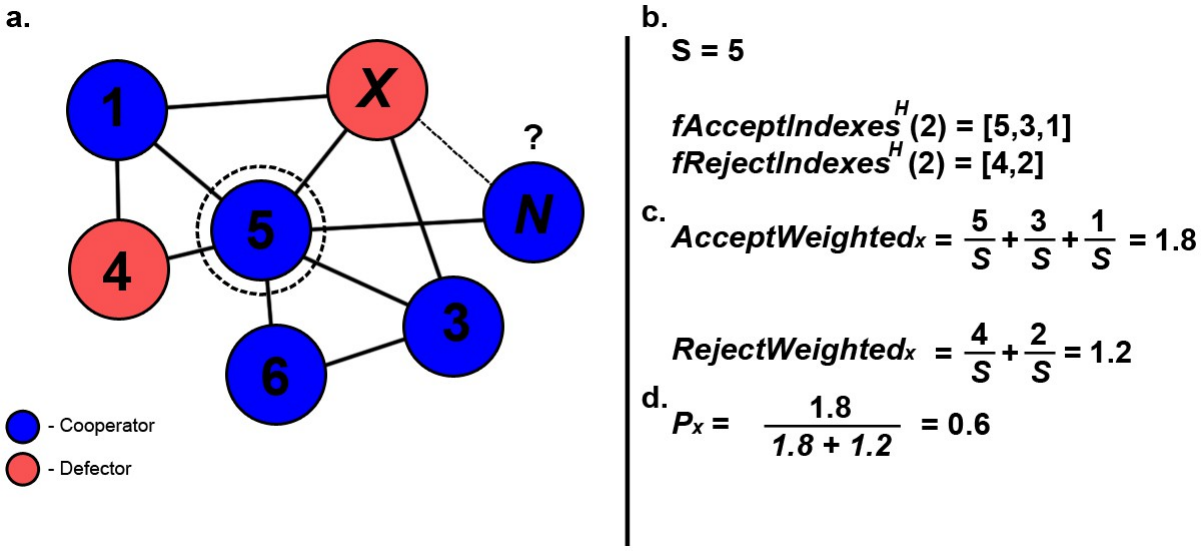
Example scenario of diminishing probabilistic aggregation method being utilised. Here, the newcomer is determining if it should form a connection with node x (a). First the indexes of accepted and rejected connections within *H* are obtained as the two defined lists (b). Next, the weights of both accepted and rejected connections are calculated by utilising the obtained index lists and the current value of *S* (c). Lastly, these values are then used to calculate *P*_*x*_, which determines the likelihood of public information indicating a connection should be made (d).

The last method to utilise global history is a variation of the previously described method we refer to as ‘Diminishing Probabilistic Aggregation’. Here the age of information is taken into account, where the latest information available to individuals has a greater relevance and indication of current trends rather than older information [22] (see Fig 6). Lists *fAcceptIndexes*^*H*^ and *fRejectIndexes*^*H*^, as described in Section 2.4, are utilised as part of (5) and (6) to calculate the values for *AcceptWeighted*_*x*_ and *RejectWeighted*_*x*_ that denote the sum of accepted and rejected connections towards node *x*, taking into consideration their position within *H* and its current size *S*. With these additional parameters, *P*_*x*_ is calculated as described in (7) and is utilised to determine the likelihood of public information indicating a connection should be formed with a node *x* (see Fig 6). As with the previous method, if the total weighted sum of accepted and rejected connections available in *H* does not exceed one, a newcomer will instead utilise a coin-toss to determine if public information will indicate a connection should be made.

Given the node *x*, public information will give an indication whether or not a connection should be formed with *x*, based on the following conditional statements:

- If *AcceptWeighted*_*x*_+ *RejectWeighted*_*x*_ > 1, then public information will indicate a connection should be formed with node *x* with Probability *P*_*x*_
- If *AcceptWeighted*+ *RejectWeighted*_*x*_ ≤ 1, then public information will indicate a connection should be formed with node *x* with Probability 0.5

$$\begin{array}{c} AcceptWeighted_{x}=\sum_{j\in fAcceptIndexes^{H}\left(x\right)}^{}\frac{j}{S} \end{array}$$ (5)

$$\begin{array}{c} RejectWeighted_{x}=\sum_{j\in fRejectIndexes^{H}\left(x\right)}^{}\frac{j}{S} \end{array}$$ (6)

$$\begin{array}{c} P_{x}=\frac{AcceptWeighted_{x}}{AcceptWeighted_{x}+RejectWeighted_{x}} \end{array}$$ (7)

### 2.4 Information based decision-making

The indications provided by private and public information are combined to determine if a newcomer will form a connection with a node *x*, which is either the role-model or one of its neighbours (Fig 1c). The indications on whether or not to establish a connection are as described in Sections 2.2 and 2.3. The indications provided by public and private information are weighted to establish the likelihood of a connection being created if only one indicates a connection with a node *x* should be made. Parameter *p* will determine the likelihood that a newcomer will form a connection when only public information indicates a connection should be made. Parameter *q* governs a similar likelihood for when only private information indicates a connection should be made. Parameters *p* and *q* are modified throughout simulations to assign more weight to one source of information over the other.

Given the node *x*, the chosen role-model or a neighbour of the chosen role-mode and the indications from public and private information, the newcomer decides whether to connect with a node *x* in the following way:

- If both public and private information indicate a connection should be made, form connection with node *x*
- If both public and private information indicate a connection should not be made, reject connection with node *x*
- If public information indicates to make a connection but private information does not, a connection with node *x* will be created with probability *p*
- If private information indicates to make a connection but public information does not, a connection with node *x* will be created with probability *q*

Once a newcomer has determined which connections it will form (see Fig 1c), the network is then updated. Following this, an individual is randomly selected to be replaced by the newcomer, keeping the population sized fixed as in the Moran process [6]. Parameter *N* sets the number of nodes present in the network. Parameter *E* sets the initial number of connections that are present in the network. Throughout simulations, we assume that the starting conditions of networks will utilise *N* = 100 and *E* = 200 with the structure being generated as a random graph. When calculating fitness, we assume *δ* = 0.001.

### 2.5 Network metrics

We study the dynamics of the model using several metrics. Cooperation is used to determine the average number of cooperators that are present on the network during a simulation. Fitness is used to determine the average level of fitness of individuals present during within a simulation. This value is normalised based on the maximum possible average fitness. Connections is used to determine the average number of connections individuals had during simulations, which is also normalised based on the maximum number of connections an individual can possess (*N*−1). As simulations progress, the choices made by newcomers are evaluated, which includes if they either correctly accept a connection with a cooperator or correctly reject a connection with a defector. If a newcomer correctly forms a connection with a cooperator, this will constitute a *True* *Positive* (*TP*); if a newcomer correctly rejects a connection with a defector, this will constitute a *True* *Negative* (*TN*); if a newcomer incorrectly forms a connection with a defector, this will constitute a *False* *Positive* (*FP*); if a newcomer incorrectly rejects a connection with a cooperator, this will constitute a *False* *Negative* (*FN*). To evaluate individual ability to differentiate between cooperators and defectors, specificity and sensitivity metrics are utilised. Specificity is considered as Eq (8) and sensitivity is considered as Eq (9). Specificity will determine how well newcomers are able to identify defectors and sensitivity indicates similarly for cooperators. As this model utilises randomly initialised networks in conjunction with the Moran process [6], these metrics will be logged during simulations following the first 10^4^ steps.

$$\begin{array}{c} TN/(TN+FP) \end{array}$$ (8)

$$\begin{array}{c} TP/(TP+FN) \end{array}$$ (9)

Another metric that is recorded during simulations is the number of transitions that occurred. Within the scope of this model, a transition is considered to have occurred when a previously absent strategy emerges as a consequence of mutation and spreads across the network to the point that it is the only remaining strategy. Should this value be large, this will indicate that a network configuration is somewhat unstable.

**Fig 7:**
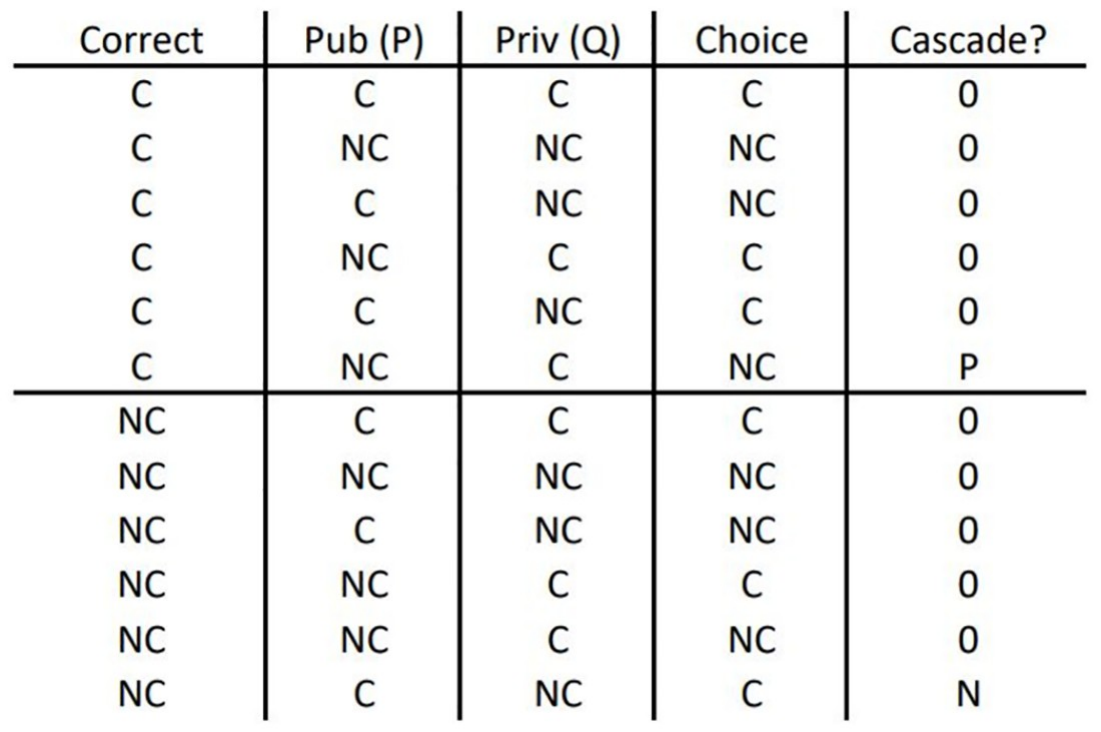
Truth table for determining which combination of choices made by newcomers are considered as contributing to cascades. The first column indicates whether the choice to connect (C) or not connect (NC) is correct. The second and third columns show the indications from public and private information. The forth column is the actual choice made by the newcomer. The fifth column indicates if a cascade has occurred, either P or N cascade, or if none has occurred with 0.

**Fig 8:**
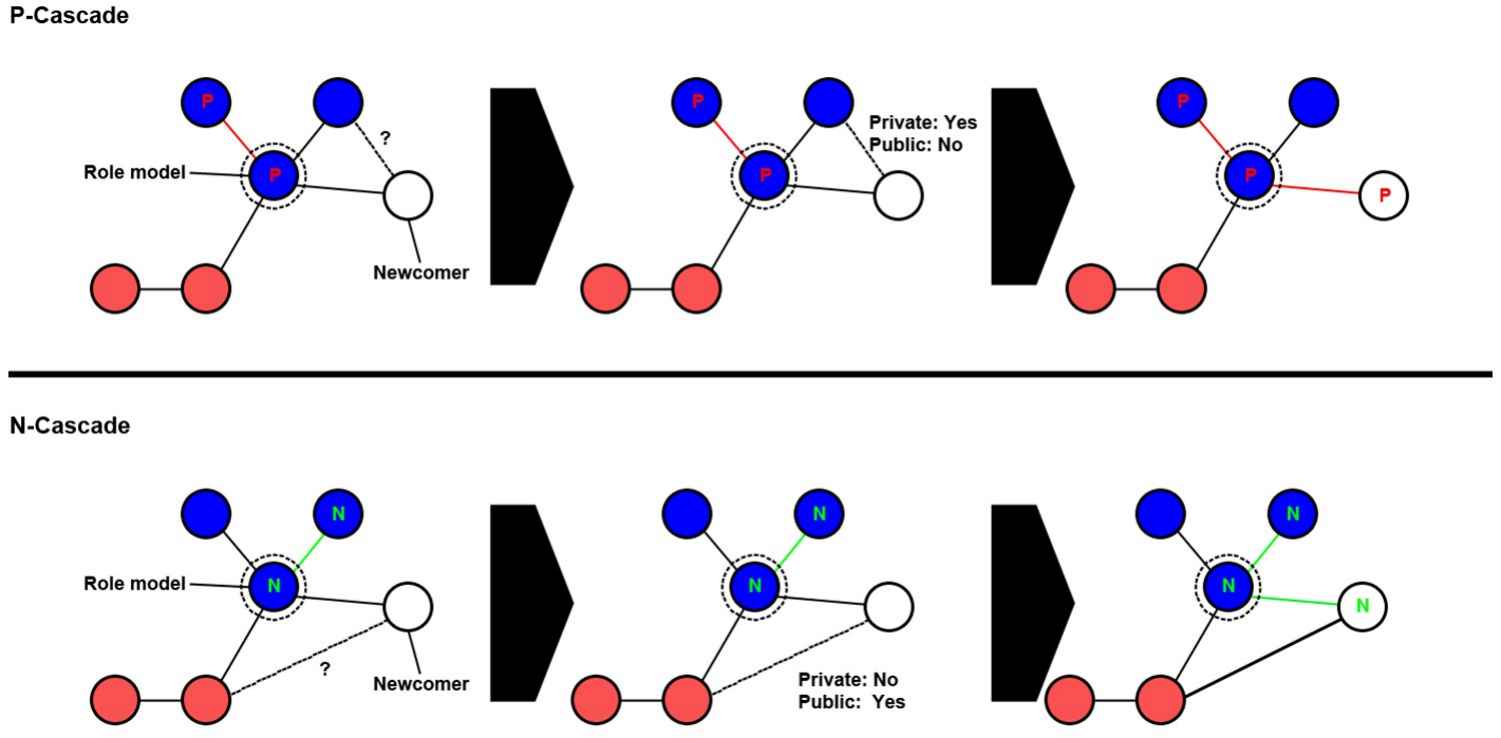
An illustration of the two types of cascades that are monitored and and how they can potentially propagate as newcomers join the network. A *P* cascade is considered to have occurred when a node does not form a connection with another cooperator based on misleading public information. A *N* cascade is considered to have occurred when a node incorrectly forms a connection with a defector based on misleading public information.

With both private and public information implemented into the model, a conflict between differing indications can arise, which can lead to erroneous decisions to be taken by individuals [17]. As described above, parameters *p* and *q* are utilised to determine the likelihood of a connection being formed when only public or private information indicates a connection should be formed, respectively. This potentially means that a conflict between public and private information may result in a newcomer making an incorrect decision when forming connections, either incorrectly rejecting a connection with a cooperator or forming a connection with a defector. As newcomers continue to join the network during simulations, these incorrect decisions can potentially propagate to other newcomers as they form connections based on their choice of role-model (see Fig 8). During simulations, the spread of these erroneous decisions are monitored as information cascades [17]. Here, we observe the emergence of two different kinds of information cascades. Illustrations of these cascades can be examined in Figs 7 and 8. The first kind of cascade that is monitored is the *P* cascade. Within the model, a *P* cascade is considered to have occurred when a newcomer rejects a connection with a potential cooperative neighbour when only private information indicates the connection should have been made and public information indicates a connection should not be made. The other kind of cascade that is monitored is the *N* cascade. An *N* cascade is considered to have occurred when a newcomer forms a connection with a defector when only private information indicates that the connection should have been rejected and public information indicates a connection should be made. For both types of cascades, the number of cascades and their length are recorded, which is calculated by determining the number of nodes that are present as part of the cascade (Figs 7 and 8). An increase in these information cascades will indicate that more newcomers are making incorrect decision when forming connections due to a conflict between public and private information.

## 3 Results

To evaluate how the different methods used to consider global history can effect cooperation and network stability, several series of simulations are carried out. In order to gain a reasonable insight into how certain network configurations can affect networks, all simulations will run for a total of 10^8^ steps each. These simulations are split into four different groups, based on the method currently utilised for public information, and are further grouped based on the balance between private and public information. Firstly, simulations are carried out where public information is based on the number of connections a node *x* possesses (see Section 2.3.1), this will provide some grounding to compare the results of the methods utilising global history against. Following on from this, the additional methods for public information, as described in section 2.3.2, are utilised as part of the decision making process, which are then analysed to determine how individual decisions based on global history can affect cooperation in networks. During these later simulations, the level of available information *S* is increased in increments of 10, with a minimum of 10 and a maximum of 50.

### 3.1 Connection average

For the first series of simulations, newcomers only utilise the current connection average of a node *x* to serve as public information, as described in Section 2.3. As *S* does not need to be adjusted for these simulations, each network configuration will only consist of one series of simulations each.

**Fig 9:**
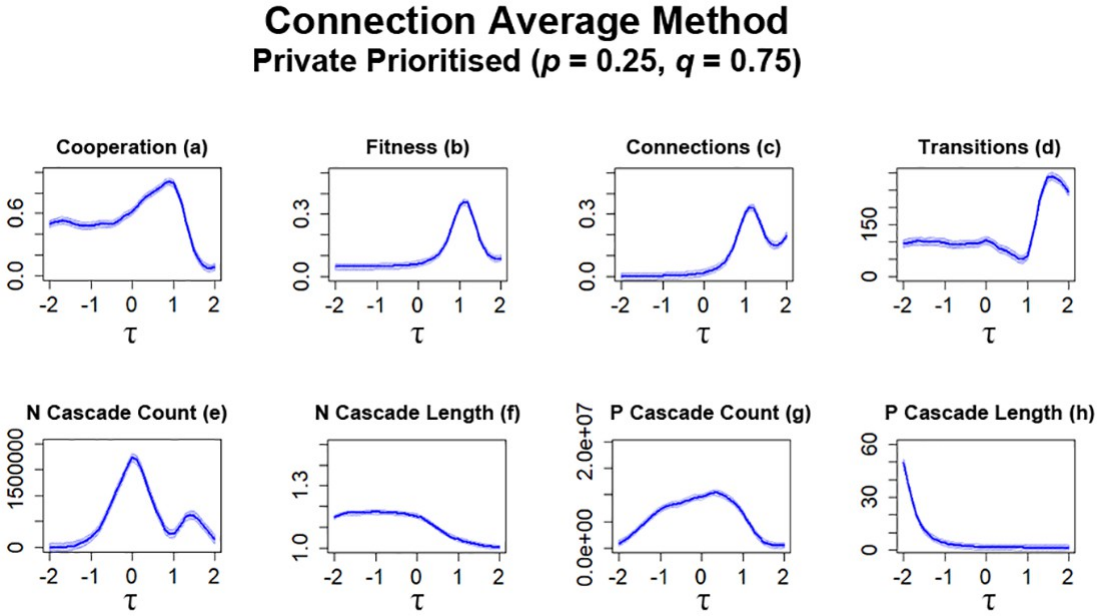
Overall cooperation that occurs in networks gradually increases as *τ* is increased in networks. However, a collapse in cooperation eventually occurs, along with a sharp increase in network transitions. The plots visualise and compare the metrics (see Section 2.5) of cooperation, fitness, average connectivity, number of transitions and the count and average lengths of information cascades that occurred. Data obtained by running the model for 10^8^ steps utilising connection average as the method for public information where private information is prioritised. *τ* represents the decision threshold. Shading represents level of standard error calculated for each dataset. Data interpolated utilising RStudio [23]. Plots produced via RStudio.

The first set of these simulations utilises private information as the primary indication for if a connection should be formed (*p* = 0.25, *q* = 0.75). As can be observed in Fig 9a, the average level of cooperation gradually increases as *τ* is increased, which aligns with what has been observed in previous works [17, 21]. However, further increasing *τ* past a value of 1 eventually results in a collapse of average cooperation (Fig 9a). This spike in cooperation aligns with what can be observed for the level of fitness (Fig 9b) with significant increase followed by a sharp decline as *τ* continues to increase. Also aligning with these observations (Fig 9a and 9b) is the number of connections formed between individuals (Fig 9c) with a sharp increase in overall connections for greater values of *τ*. Something to note is the sharp increase in the number of transitions (Fig 9d) which occurs as a sharp decline in cooperation occurs (Fig 9a). With public information also present within these networks, the occurrence of information cascades are also recorded during simulations. There is a spike in *N* cascades for simulations with *τ* ≈ 0 and are generally short in length (Fig 9e and 9f). *P* cascades occur significantly more than *N* cascades throughout simulations (Fig 9g) and are generally quite long in length for lower values of *τ* (Fig 9h).

**Fig 10:**
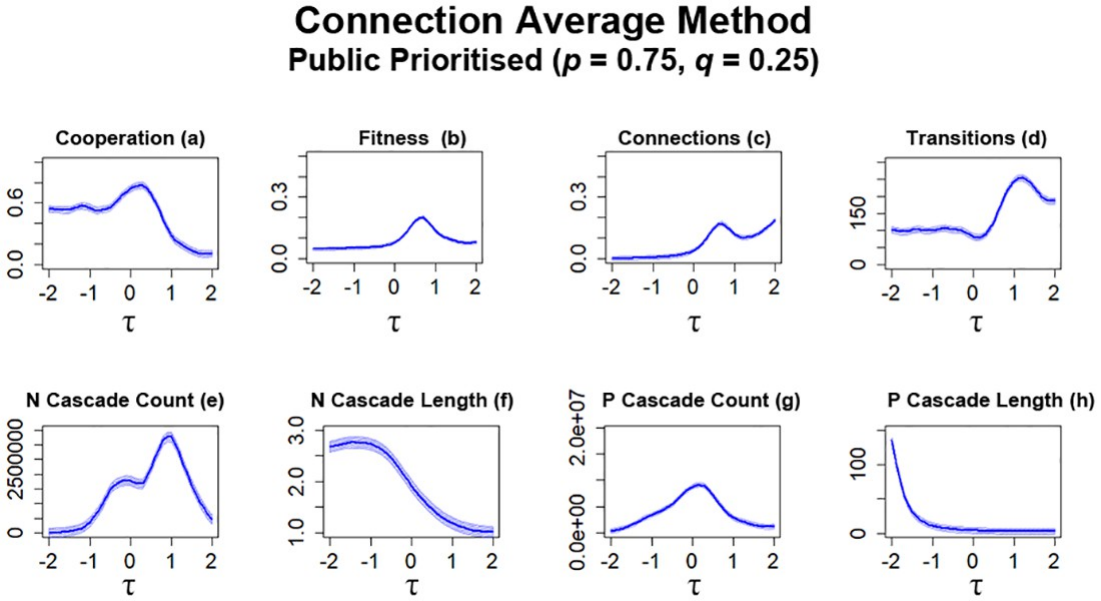
Prioritising public information leads to an overall decrease in cooperation occurring in networks. The plots visualise and compare the metrics (see Section 2.5) of cooperation, fitness, average connectivity, number of transitions and the count and average lengths of information cascades that occurred. Data obtained by running the model for 10^8^ steps utilising connection average as the method for public information where public information is prioritised. *τ* represents the decision threshold. Shading represents level of standard error calculated for each dataset. Data interpolated utilising RStudio [23]. Plots produced via RStudio.

For the next series of simulations, nodes are configured to prioritise public information over private information (*p* = 0.75, *q* = 0.25). With these configurations, some network metrics are largely similar to simulations where connection average was utilised (Fig 9) with some slight decreases. The average level of cooperation present in networks is largely similar, but the greatest point in cooperation is noticeably lower than simulations prioritising private information (Fig 10a). The level of observed fitness is also noted to be somewhat lower (Fig 10b) than simulations prioritising private information (Fig 9b), following similar observations noted in previous works [17]. With public information prioritised, the number of connections formed between individuals is also impacted, with overall less connections being formed (Fig 10c). Although the number of transitions still increases for higher values of *τ* (Fig 10d), this peak is somewhat more subdued than simulations prioritising private information (Fig 9d). Prioritising public information also leads to some differences in the occurrences of information cascades (Fig 10). When compared to simulations prioritising private information (Fig 9), the number of *N* cascades that occur generally increase, particularly at higher values of *τ* (Fig 10e). The average lengths of these *N* cascades are also significantly greater (Fig 10f) than previously recorded occurrences (Fig 9b). For *P* cascades, there is an overall decrease in occurrences, with a peak in *P* cascades occurring for *τ* ≈ 0 (Fig 10g). There is also a marked increase in average length for *P* cascades at lower values of *τ* (Fig 10h).

### 3.2 Bikchandani method

We investigate the effects of the different methods that use global history in the decision-making process and how they can affect cooperation within networks. For this series of simulations, newcomers utilise the ‘Bikchandani’ method as it is described in Section 2.3.2. As previously described (Section 3), *S* is initially be set as 10 and increased in increments of 10, up to a maximum of 50. We remind that *S* represents that maximum number of entries that can be stored within *H*, i.e. the amount of public information available to newcomers. As with simulations utilising connection-average, the values of *p* and *q* are altered to adjust the balance of public and private information throughout simulations (see Section 2.4).

**Fig 11:**
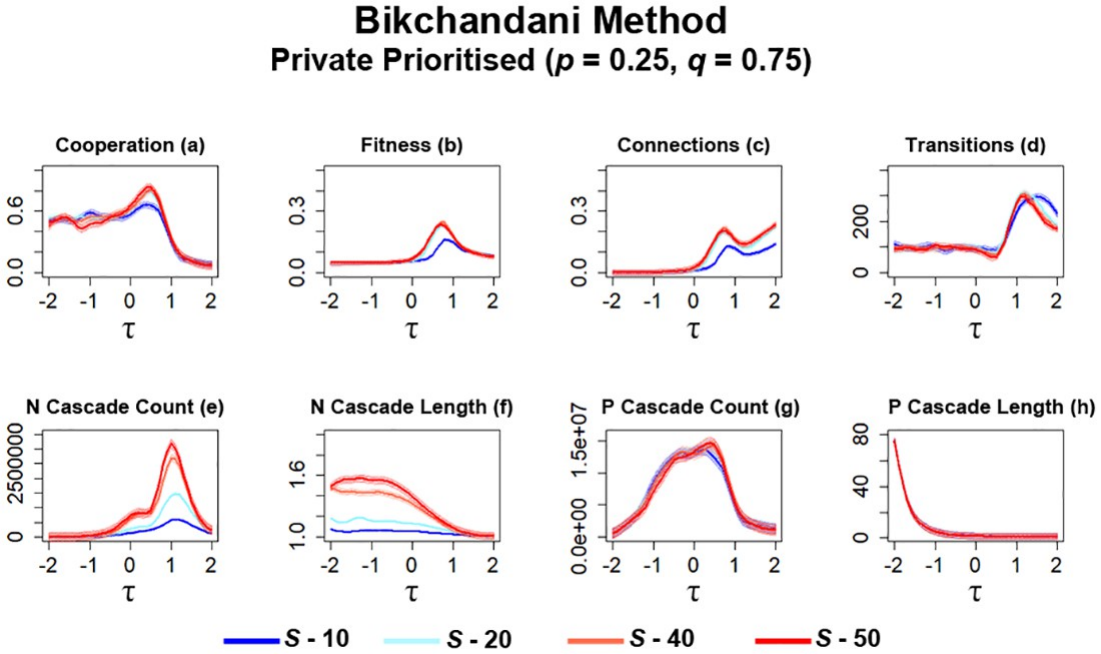
Networks utilising global history are able to generate more cooperation and connections amongst individuals as more information is made available by increasing *S*, which comes at a cost of increased information cascades. The plots visualise and compare the metrics (see Section 2.5) of cooperation, fitness, average connectivity, number of transitions and the count and average lengths of information cascades that occurred. Data obtained by running the model for 10^8^ steps utilising the ‘Bikhchandani’ method for public information where private information is prioritised. *τ* represents the decision threshold. *S* represents the number of entries present in global history *H*. Shading represents level of standard error calculated for each dataset. *S* represents the number of entries present in global history *H*. Shading represents level of standard error calculated for each dataset. Data interpolated utilising RStudio [23]. Plots produced via RStudio.

The first series of simulations utilising the ‘Bikchandani’ method is carried out in networks where private information is prioritised over public information (*p* = 0.25, *q* = 0.75). Examining data from these simulations shows that both the value of *S* and how information in *H* is utilised by individuals can affect the level of cooperation occurring in networks (Fig 11a). Firstly, increasing the value of *S* in general leads to an increase in cooperation present in networks (Fig 11a). Although, when compared to simulations utilising connection average (Fig 9a), there is a general decrease in the level of cooperation, especially for lower values of *S* (Fig 11a). A similar trend can also be observed when examining the average fitness that occurs within these networks (Fig 11b). Increasing *S* leads to an increase in fitness, with the eventual spike aligning just before the eventual collapse in cooperation for higher values of *τ* (Fig 11a). Something to note here is that there comes a point where increasing *S* can be detrimental to fitness. Networks set as *S* = 40 are able to attain a higher point of fitness than networks where *S* = 50 (Fig 11b). One of the most significant changes with this method is the change in the number of connections formed between individuals (Fig 11c). Overall, the average number of connections is significantly lower when compared to connection average simulations (Fig 9c), which may suggest that individuals are less open to forming connections with others when historical information is available to them rather than limiting their observations of others to a specific point in time and just considers well-connected individuals (see Section 2.3.1). Similarly, lower values of *S* further decreases the number of connections formed, suggesting that individuals are also less likely to form connections when little information is available (Fig 11c). Lastly, similar to observations regarding fitness (Fig 11b), setting *S* = 50 slightly impacts the number of connections formed when compared to simulations set as *S* = 40, which aligns with observed fitness. Utilising this method also has some effect on the number of transitions that occurred (Fig 11d). At higher values of *τ*, increasing *S* leads to some decreases for the number of transitions occurring in networks.

When compared against simulations utilising connection average (Fig 9d), the number of transitions overall decreased when this method is utilised, which may suggest that network stability may be more viable as individuals are enabled to make more nuanced decisions when observing others in networks. One of the more prominent areas where the effects of this information method could be observed is in the occurrences of information cascades (Fig 11), mainly *N* cascades. When the ‘Bikchandani’ method is utilised by networks, the occurring spike in *N* cascades occurrences shift towards higher values of *τ* (Fig 11e), rather than centered towards 0 in networks utilising the connection average method (Fig 9e). As *S* is increased, the occurrences in *N* cascades significantly increased, with a difference in average length also noted (Fig 11f). These observations suggest that newcomers are more likely to form connections with defectors when pubic information indicates a connection should be made, which aligns with how this method functions as the indications of private information can be overwritten by the indication of public information (see Section 2.3.2). Depending on the value of *S*, networks can either exceed or not exceed the number of *N* cascades occurrences observed during connection average simulations (Fig 9e). Some notable changes in *P* cascades are also noted here. As information availability increases, there is a slight increase in *P* cascades at *τ* ≈ 0.5 (Fig 11g) and no significant difference in average length (Fig 11h). However, when compared to connection average simulations (Fig 9c and 9d), significantly less *P* cascades occurred overall when newcomers utilised the Bikchandani method when evaluating potential neighbours.

**Fig 12:**
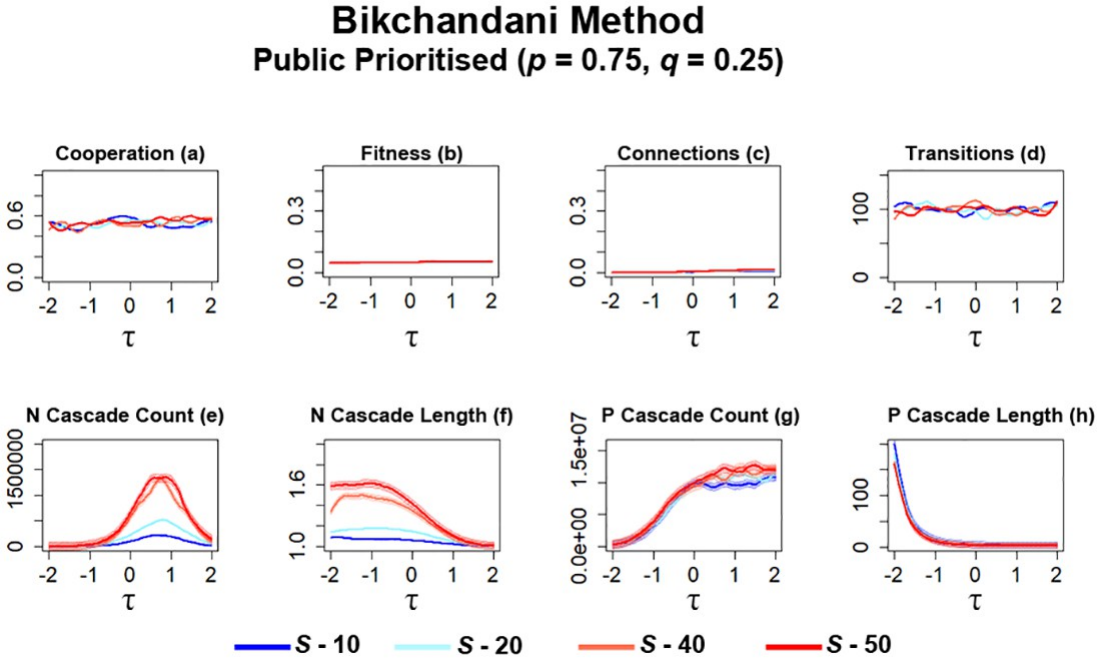
Cooperation in networks stagnate when public information is prioritised whilst utilising the ‘Bikhchandani’ method in networks regardless of the value of *S*. The plots visualise and compare the metrics (see Section 2.5) of cooperation, fitness, average connectivity, number of transitions and the count and average lengths of information cascades that occurred. Data obtained by running the model for 10^8^ steps utilising the ‘Bikhchandani’ method for public information where public information is prioritised. *τ* represents the decision threshold. *S* represents the number of entries present in global history *H*. Shading represents level of standard error calculated for each dataset. Data interpolated utilising RStudio [23]. Plots produced via RStudio.

For the next series of simulations, networks are configured to prioritise public information over private information (*p* = 0.75, *q* = 0.25) when newcomers determine which potential neighbours they should form connections with. In previous works [17, 21] and simulations utilising connection average (Fig 10), prioritising public information leads to some changes in network metrics, although they still largely resemble trends that occur in networks that prioritise private information (Fig 9). However, in networks where the ‘Bikchandani’ method is utilised, the result is stagnation, which we consider here as no significant increases or decreases in cooperation and connections, across all networks that utilise different *τ* values (Fig 12).

Cooperation in these networks (Fig 12a) results in an average level of ≈0.5 and networks are able to avoid a sharp collapse in cooperation regardless of the current value of *τ*. The trade off to this is that no networks are able to generate higher levels of cooperation. This observation aligns with the observed levels of fitness in these networks (Fig 11b). Under these conditions, there is no significant difference in fitness, networks with higher values of *S* are only able to attain a slightly higher level of fitness. The number of transitions that occur also stagnate (Fig 12d), suggesting that both strategies had a difficult time propagating throughout networks under these conditions. Examining observed connections may offer some reasoning behind these observations (Fig 12c). Whilst networks with greater information availability results in slightly more connections, all networks under these configurations result in very few connections being made between individuals, especially when compared against simulations utilising connection average (Fig 10c). Connection average also gradually increases as *S* is increased, suggesting that newcomers become more open to forming connections with more information via *S*, although they are still severely limited when prioritising public information. Given the rules defined for the ‘Bikchandani’ method in section 2.3.2, these observations suggest that when prioritising public information, newcomers are far less likely to form connections due to a entries in *H* encouraging the formation of more connections. This possibly resulted in more rejected connections and this resulting information may have further dissuaded later newcomers from forming connections.

In simulations utilising connection average (Fig 10), prioritising public information leads to some increases in the number of information cascades that occur. However, when the ‘Bikchandani’ method is utilised under these configurations, a significant decrease in N information cascades occurs (Fig 12e). Increasing *S* still results in a gradual increase in the number of *N* cascades that occur within networks (Fig 12e) with their average length also gradually increasing (Fig 12f). *P* cascades, in general, also increase in occurrence as *S* is increased (Fig 12g). Although here, P cascade occurrence remains high for higher values of *τ*, unlike simulations prioritising private information where *P* cascades eventually drop off (Fig 11g). Here, it is also possible to observe some notable changes in P cascade length, with networks with higher values of *S* resulting in somewhat shorter *P* cascades (Fig 11h).

### 3.3 Probabilistic aggregation method

Following on from simulations utilising the ‘Bikchandani’ method, public information is updated to utilise the probabilistic aggregation method, as described in section 2.3.2. Like previous simulations, the amount of available information via *S* is gradually increased in increments of 10, starting at 10 up to a maximum of 50.

**Fig 13:**
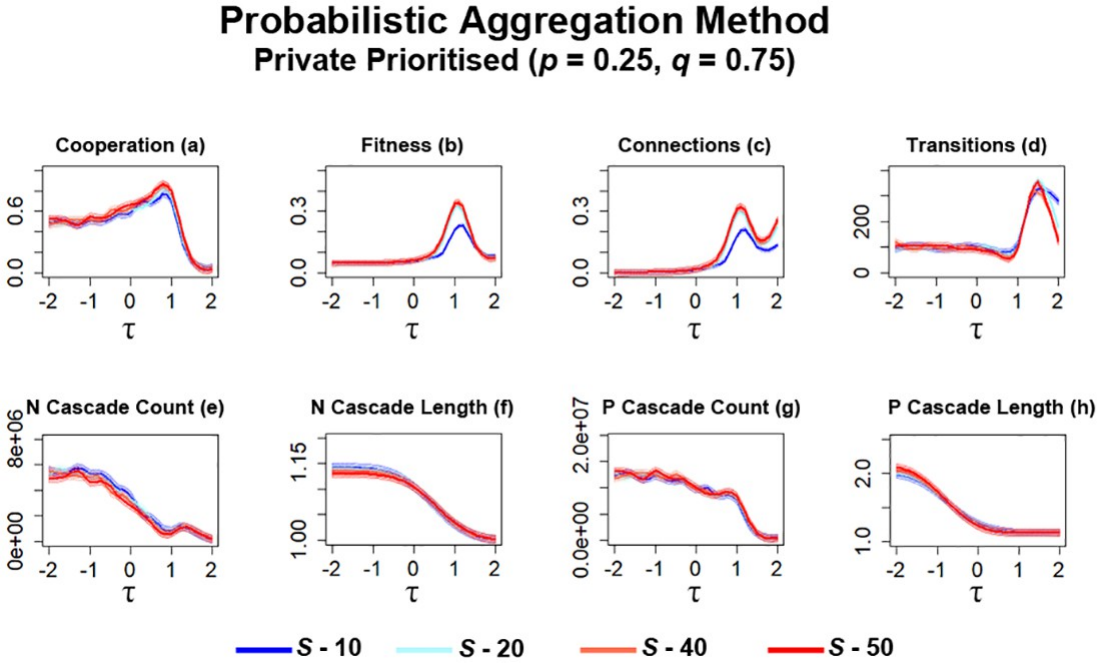
Networks generate higher levels of cooperation as *S* is increased. When public information is based on the probabilistic aggregation method and private information is prioritised, this results in a sharp increase in the number of information cascades that occur within networks. The plots visualise and compare the metrics (see Section 2.5) of cooperation, fitness, average connectivity, number of transitions and the count and average lengths of information cascades that occurred. Data obtained by running the model for 10^8^ steps utilising the probabilistic aggregation as the method for public information where private information is prioritised. *τ* represents the decision threshold. *S* represents the number of entries present in global history *H*. Shading represents level of standard error calculated for each dataset. Data interpolated utilising RStudio [23]. Plots produced via RStudio.

For the first series of simulations utilising probabilistic aggregation, networks prioritise private information over public information (*p* = 0.25, *q* = 0.75). Similar to ‘Bikchandani’ simulations (Fig 11a), the level of cooperation that occurs within networks gradually increases as *S* is increased, with networks generating slightly more cooperation (Fig 13a). Although, the difference between the highest and lowest maximum observed level of cooperation is much smaller than in the case of ‘Bikchandani’ simulations (Fig 11a). A collapse in cooperation continues to occur once *τ* exceeds a threshold of ≈1. A similar trend can be observed for the level of fitness in these networks (Fig 13b) with greater values of *S* leading to a higher level of fitness and are capable of generating a higher level of fitness than ‘Bikchandani’ simulations (Fig 11b). Networks observed with these parameters are also able to result in more connections being formed between individuals (Fig 13c) than ‘Bikchandani’ simulations (Fig 11c). The trade-off to some of these improvements is a slight increase in the number of transitions that occurred during simulations (Fig 13d) after a threshold of *τ* is crossed at ≈1, which aligns with observations regarding the collapse in cooperation (Fig 13a). Although higher values of *S* can suppress the number of transitions at certain points (*τ* ≈ 1 & *τ* ≈ 2), this does result in less cooperation being generated at the highest values of *τ* (Fig 13a).

One area where a dramatic difference occurs is with the number of information cascades observed in networks (Fig 13). The number of cascades here not only exceed connection average simulations (Fig 9) but greatly exceed the number of occurrences observed for the ‘Bikchandani’ method (Fig 11). In general, as *S* is increased, the number of *N* cascades decreases (Fig 13e). Although more *N* cascades occur under these parameters, the number of these greatly diminishes as *τ* is increased, and reaches a similar number of cascades observed in ‘Bikchandani’ simulations (Fig 11e) prior to the increase in cooperation at *τ* ≈ 1 (Fig 13a). Increasing *S* also results in slightly shorter *N* cascades occurring (Fig 13f). The number of *P* cascades that occur for lower values of *τ* exceed that of ‘Bikchandani’ simulations (Fig 11g), with similar levels of occurrence observed for higher values of *τ* (Fig 13g). The average length of *P* cascades (Fig 11h) also significantly decreases compared to ‘Bikchandani’ simulations (Fig 11h), which may be due to the increase in these cascades occurring. Increasing *S* also results in slightly longer *P* cascades at lower values of *τ* (Fig 11h).

**Fig 14:**
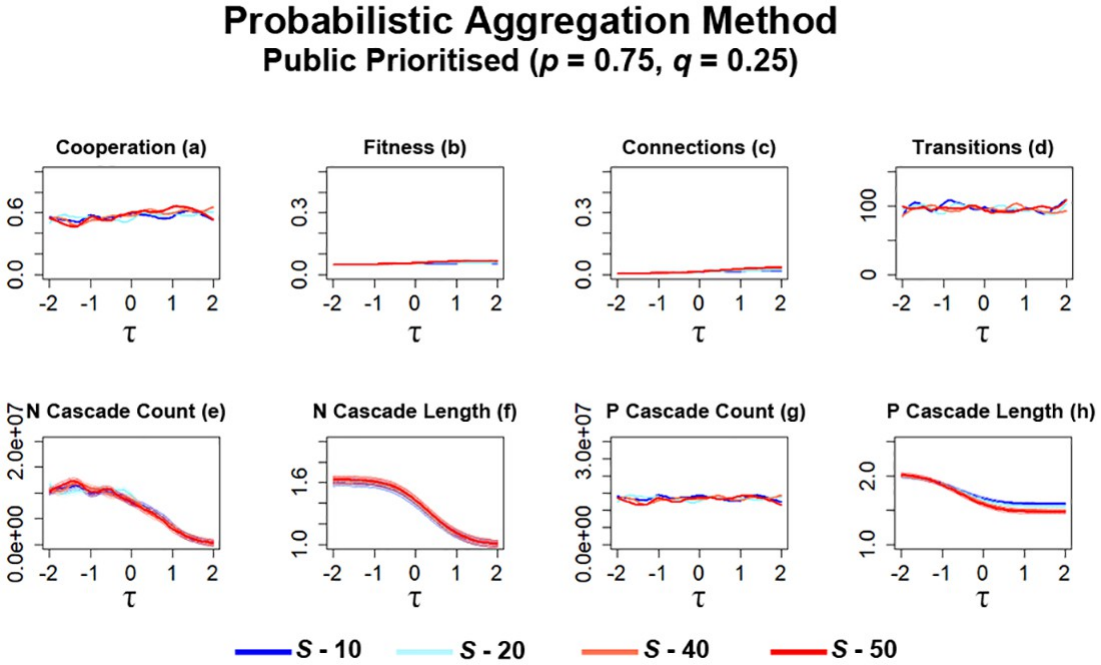
Stagnation of cooperation, fitness and stability occurs in networks utilising probabilistic aggregation when public information is prioritised over private information. The plots visualise and compare the metrics (see Section 2.5) of cooperation, fitness, average connectivity, number of transitions and the count and average lengths of information cascades that occurred. Data obtained by running the model for 10^8^ steps utilising the probabilistic aggregation as the method for public information where public information is prioritised. *τ* represents the decision threshold. *S* represents the number of entries present in global history *H*. Shading represents level of standard error calculated for each dataset. Data interpolated utilising RStudio [23]. Plots produced via RStudio.

To further evaluate the effects of probabilistic aggregation, networks are configured to prioritise public information over private information (*p* = 0.75, *q* = 0.25). Similar to what occurs with the ‘Bikchandani’ method under these conditions (Fig 12), networks here also stagnate (Fig 14). Here, networks result in a middling level of cooperation with no collapse occurring (Fig 14a). When examining values of *τ* ≈ 1, where previous peaks in cooperation tend to occur when private information is prioritised (Fig 14a), increasing *S* leads to a minor increase in cooperation. This aligns with observations regarding fitness (Fig 14b), where increased availability of information via *S* leads to a slight increase in fitness. Networks here also appear to generate slightly higher levels of fitness with additional information when compared to ‘Bikchandani’ simulations (Fig 12b). A minor increase also occurs for connection formation as *S* is increased (Fig 12c), which aligns with observations regarding cooperation and fitness. The connection count continues to remain very low under these conditions. Similar to ‘Bikchandani’ simulations (Fig 12d), the number of transitions here (Fig 14d) are relatively lower than connection average simulations (Fig 10d) and largely remain at the same level of occurrence regardless of the current value of *τ*.

Similar to simulations prioritising private information (Fig 13), the number of cascades observed continues to occur more frequently (Fig 14) than other utilised information types (Figs 10 & 12). As with simulations prioritising private information (Fig 13), *N* cascades occur most at the lowest value of *τ* and gradually decrease in occurrence as *τ* is increased (Fig 14e). Average length also gradually decreases, with networks with greater values of *S* resulting in slightly longer *N* cascades (Fig 14f). Unlike ‘Bikchandani’ simulations (Fig 12g), the occurrences of *P* cascades remain frequent throughout all simulations here regardless of both values of *S* and *τ* (Fig 14g). Increasing *S* slightly decreases the average length of *P* cascades at higher values of *τ* (Fig 14h). One noticeable change when compared to ‘Bikchandani’ simulations (Fig 12h) is that the average length of *P* cascades here is much shorter (Fig 14h), although this is likely due to an increased occurrence of these cascades at lower values of *τ*.

### 3.4 Diminishing probabilistic aggregation method

We next observe the effects of the method diminishing probabilistic aggregation, as described in section 2.3.2. As with previous simulations, *S* is initially set as 10 and is increased in increments of 10 up to a maximum of 50, the weights of information types is also adjusted throughout simulations.

**Fig 15:**
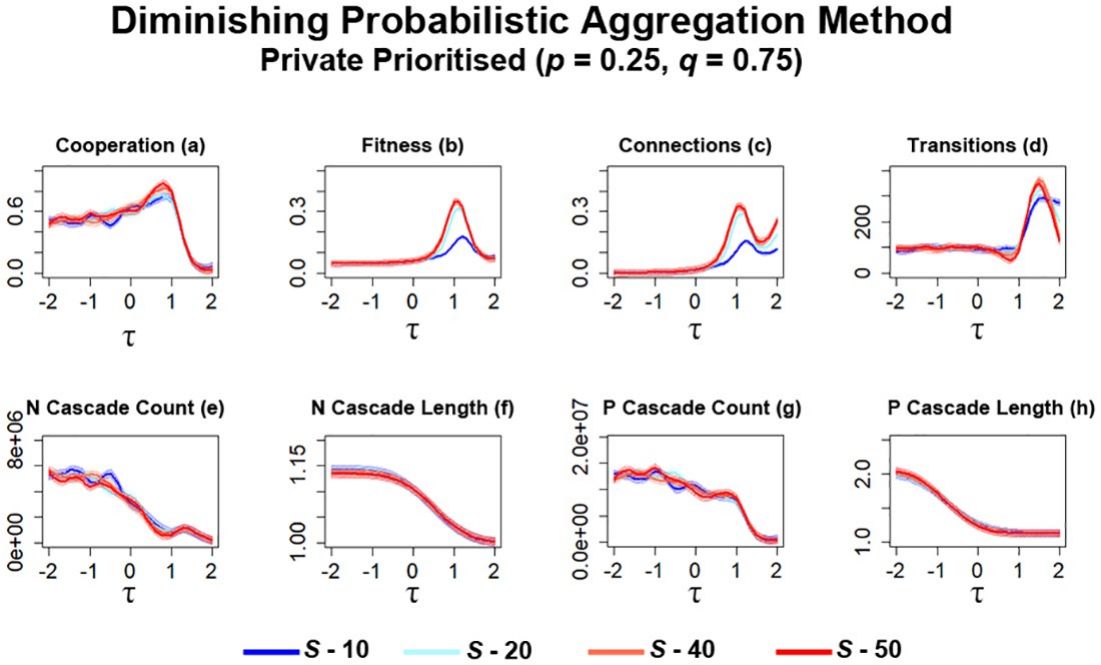
Cooperation greatly increases in networks as *S* is increased when diminishing probabilistic aggregation is utilised by networks. The plots visualise and compare the metrics (see Section 2.5) of cooperation, fitness, average connectivity, number of transitions and the count and average lengths of information cascades that occurred. Data obtained by running the model for 10^8^ steps utilising the Probabilistic Aggregation as the method for public information where private information is prioritised. *τ* represents the decision threshold. *S* represents the number of entries present in global history *H*. Shading represents level of standard error calculated for each dataset. Data interpolated utilising RStudio [23]. Plots produced via RStudio.

Simulations here begin with networks that prioritise private information over public information (*p* = 0.25, *q* = 0.75). Similar to simulations utilising probabilistic aggregation (Fig 13a), the level of cooperation observed in these networks gradually increases as *S* is increased (Fig 15a). Compared with probabilistic aggregation (Fig 13a), networks attain a higher average of cooperation for higher values of *S*, whilst networks utilising lower values of *S* attain a lower average of cooperation (Fig 15a). The level of fitness that occurs here (Fig 15b) follows similar patterns to probabilistic aggregation simulations (Fig 13b) with increasing *S* leading to an increase in fitness. However, in this case, there is a greater decrease here in fitness for networks that utilise the least amount of information to work with (Fig 15b). Networks continue to garner an increase in connectivity between individuals as *S* is increased (Fig 15c), similar to previously observed patterns (Fig 13c). A decrease in connections formed can be observed for networks utilising little information (Fig 15c), which aligns with observations regarding the changes in both cooperation and fitness (Fig 15a and 15c). Increasing *S* in general leads to an increase in transitions occurring (Fig 15d), although observations suggest there comes a point where enough information can start to decrease the number of transitions within these networks, particularly for *τ* ≈ 0.8. The difference between these observed transitions (Fig 15d) also increases when compared with probabilistic aggregation (Fig 13d).

The observations regarding information cascades (Fig 15) largely resemble that of simulations utilising probabilistic aggregation (Fig 13). Compared with connection average simulations (Figs 9 and 11), a significant increase in both *N* and *P* cascades occur within these networks, particularly for lower values of *τ*. Networks that utilise lower values of *S* appear to generally result in more *N* cascades occurring with a slight increase in length (Fig 15e and 15f). Findings from these networks also suggest that in some cases, increasing the amount of information available to newcomers can lead to increases in *P* cascades (Fig 15g), particularly at *τ* ≈ −1 and *τ* ≈ 1. Increasing *S* also results in slightly longer *P* cascades occurring (Fig 15h).

**Fig 16:**
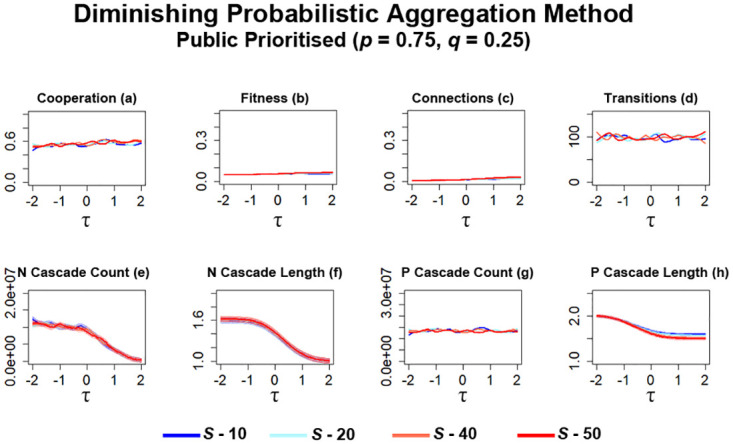
Stagnation occurs when public information is prioritised whilst utilising diminishing probabilistic aggregation. The plots visualise and compare the metrics (see Section 2.5) of cooperation, fitness, average connectivity, number of transitions and the count and average lengths of information cascades that occurred. Data obtained by running the model for 10^8^ steps utilising the probabilistic aggregation as the method for public information where public information is prioritised. *τ* represents the decision threshold. *S* represents the number of entries present in global history *H*. Shading represents level of standard error calculated for each dataset. Data interpolated utilising RStudio [23]. Plots produced via RStudio.

Further simulations are then carried out where networks prioritise public information (*p* = 0.75, *q* = 0.25) whilst utilising diminishing probabilistic aggregation. As with networks utilising other information types (Figs 12a & 14a), networks stagnate with no collapse or significant increases of cooperation occurring (Fig 16a). When compared to probabilistic aggregation simulations (Fig 14a), there is little difference in cooperation between networks that utilise different values of *S* (Fig 16a). Little difference in the level of fitness can also be observed in these networks, with only a slight increase for networks that utilised greater values of *S* (Fig 16b). Fitness here still remains very low compared to simulations where private information was prioritised (Fig 15b). The number of connections formed between individuals also decreases slightly (Fig 16c) which may offer some reasoning behind some of the changes observed for cooperation and fitness (Fig 16a and 16b). As with simulations utilising ‘Bikchandani’ and probabilistic aggregation methods under these conditions (Figs 12d & 14d), transitions remain low with no significant difference between networks utilising differing levels of information occurring (Fig 16d).

The occurrences of information cascades here (Fig 16) largely resemble the patterns observed in probabilistic aggregation simulations (Fig 14) with only minor differences. Similar to probabilistic aggregation simulations (Fig 14e), the majority of *N* cascades occur at lower values of *τ* (Fig 16e) with no significant difference between networks utilising different values of *S*. Overall, the occurrences of *N* cascades appear to have decreased (Fig 16e) when compared to probabilistic aggregation simulations (Fig 14e). The average length of these *N* cascades remain fairly short (Fig 16f). No significant changes to the occurrences of P cascade (Fig 16g) can be noted when compared against the previous method of information evaluation (Fig 14g), with their average length also remaining fairly short (Fig 16h), although a slight increase in length can be observed for networks utilising more information via *S*.

## 4 Discussion

By utilising a novel computational simulation model, we have been able to observe the changes that occur within networks when newcomers are able to observe the decisions of previous newcomers. We show that the amount of information available to individuals and how newcomers interpret that information can influence the dynamics of the networks, and, in particular the amount of cooperation, prosperity and instability of the population.

One of the general aspects that could be observed when global history is present is that cooperation, fitness and networks connectivity increases as more information (controlled by the parameter *S*) is made available to newcomers (Figs 11, 13 & 15). This suggests that when global history is part of the decision-making, cooperative individuals are more open to forming connections with other cooperative nodes when a sufficient amount of information is available (Figs 11, 13 & 15). However, in general, when global history is employed, the amount of cooperation in the population is generally lower (Figs 11, 13 & 15) than in the case of decision-making just based on the connection average method (Fig 9) where cooperative individuals were more likely to form connections with each other, even though this method is restricted to examining the present state of the network. Alongside these observations, the number of information cascades sharply increase when newcomers utilise global history as part of their decision-making (Figs 11, 13 & 15). These results suggest that as global history is utilised, cooperative individuals become more prone to making incorrect connection decisions and also more reluctant to form connections, which may have contributed to decrease in cooperation. This may be occurring due to allowing newcomers to also consider rejected connections with global history rather than being restricted to only considering accepted connections with the connection average method.

The networks connectivity is also affected with less, when the global history is part of the newcomer decision-making, individuals may be more reluctant to form connections with others than in the case of employing the connection average method and ignoring previous decisions, particularly rejected connections (see Fig 9).

When the relevance of previous decisions is weighted according to their “age” (Figs 13 & 15), we have observed that, for higher values of *S*, networks are able to attain a higher level of fitness (Fig 15b) than in the scenario where all previous decisions are just equally weighted, irrespective of their timing (Fig 13b). However, the inverse of this can be observed with lower values of *S* resulting in lower fitness when the age of information is considered (Fig 15b) than in the scenario where previous decisions are all equally considered (Fig 13b). This last observation suggests that when newcomers account for more recent information as part of their decision-making, the amount of information available to newcomers can have a more pronounced effect upon the fitness of individuals (Fig 15b), the formation of new connections (Fig 15c) and the emergence of transitions (Fig 15d).

Decision-making based on the probabilistic aggregation method or the diminishing probabilistic aggregation method also results in an increase of cooperation and fitness, compared to the ‘Bikchandani’ method, which, however, comes at the cost of higher instability, as we can see in Figs 11, 13 and 15.

A significant change in the emergence of information cascades can also be observed when newcomers utilise the ‘Bikchandani’ method (Fig 11) than in the scenario of the probabilistic aggregation method (Fig 13) (see Section 2.3.2). A sharp increase in the number of information cascades occurs as probabilistic aggregation is utilised (Fig 13) when compared against the scenario corresponding to the ‘Bikchandani’ method (Fig 11). This could also be observed for diminishing probabilistic aggregation (Fig 15). These results suggest that when information is used in a less decisive manner (see Section 2.3.2), this can potentially contribute to a significant increase in the occurrences of information cascades.

These increases appear to largely occur at lower values of *τ*, when connections between individuals are significantly lower (Figs 11, 13 & 15). The intuition is that, in this case, the information regarding accepted connections will be very limited due to a lack of connections being formed and therefore less “encouraging” information, suggesting to form connections, will be available to newcomers. In general, utilising global history (Figs 11, 13 & 15) results in a significant increase in the number of information cascades that occur when compared against the cascades observed when the decision-making is based on the connection average method that does not consider previous decisions (Fig 9). Although, the average lengths of cascades when the decision-making uses the global history are significantly shallower (Figs 11, 13 & 15).

Also, the presence of global history in the decision-making has important consequences when public information is prioritised over private information (Figs 12, 14 & 16). Under these conditions, when the decision-making employs the global history and regardless of the value of *S*, general stagnation with no collapses in cooperation occurs within for these networks (Figs 12, 14 & 16). When previous decisions are not considered (i.e., connection average method) for public information, there is a an overall higher level of cooperation and more connections (Fig 10a and 10c). However, this comes at the cost of more instability with an increase in the number of transitions (Fig 10d). These results also illustrate the importance of private information when global information is utilised in the decision-making and they suggest that without an initial push from private information, the population results in reluctant individuals less likely to form connections, adding more rejected decisions to the recorded global history, and therefore further dissuading future newcomers from forming connections.

Several future extensions of this work can be highlighted. All newcomers in the presented model utilise the same method of interpreting information available to individuals. In a previous work [21], it has been considered the case where private opinion can vary amongst individuals. It’s likely that individuals in scenarios similar to what is simulated here will interpret information in their own way and may also be influenced by other criteria such as their past experiences or individual preferences. An individual with greater ‘social-capital’ or differing methods of sharing information with others may also have an influence on the decision-making undertaken by others, which may warrant further consideration [24, 25]. Further study should be considered to examine additional network metrics for selected role-models to identify when they are likely to have greater payoffs within a network and therefore are more likely to be chosen as role-models. It may also warrant further study to consider additional methods of interpreting both the current connection count and changes to connections over a period of time for individuals within networks. For example, newcomers could utilise eigenvector centrality rather utilising a binary choice based on the current connection count of an individual. It is also likely that information can also be misinterpreted or ignored by individuals during decision-making, which also further raises questions regarding scenarios where information shared with others can be either incorrectly or knowingly falsified by those sharing it [26–28]. An individual may also consider the reputation of another individual providing some information, both in terms of the actions they have previously undertaken and the validity of information they have previously shared with the network. It may warrant further research to explore these points to further understand how the presence of information such as this can have an impact on cooperation, particularly in scenarios where the impact of defection may not be necessarily limited to an individual’s local group [29, 30].

Overall, the presented results illustrate some of the potential effects that both varying the amount of available information to individuals and how those individuals evaluate this information can have on cooperation in dynamical networks. The findings highlight that the way the previous decisions are taken into account can influence both the well-being of these networks, their cooperation and as well as the emergence of information cascades, particularly when using all of the available information rather than just examining the difference in previous accepted and rejected connections. As part of future work, it may be worth exploring other methods of evaluating previous decisions, as well as the possibility of considering networks where individuals could choose to interpret the previous decision in an non-homogeneous way [21, 27, 28].

## References

[1] Brodie B. Strategy in the Missile Age. Princeton University Press; 2015.

[2] Levin S. Crossing Scales, Crossing Disciplines: Collective Motion and Collective Action in the Global Commons. Philosophical Transactions of the Royal Society B: Biological Sciences. 2010;365(1537):13–18. doi: 10.1098/rstb.2009.0197 20008381 PMC2842704

[3] Tappin BM, Pennycook G, Rand DG. Thinking Clearly About Causal Inferences of Politically Motivated Reasoning: Why Paradigmatic Study Designs Often Undermine Causal Inference. Current Oopinion IN Behavioral Sciences. 2020;34:81–87. doi: 10.1016/j.cobeha.2020.01.003

[4] Hauser OP, Hendriks A, Rand DG, Nowak MA. Think global, act local: Preserving the global commons. Scientific Reports. 2016;6(1):36079. doi: 10.1038/srep36079 27808222 PMC5093714

[5] Aviles L. Cooperation and non-linear dynamics: an ecological perspective on the evolution of sociality. Evolutionary Ecology Research. 1999;1(4):459–477.

[6] Nowak MA. Evolutionary Dynamics: Exploring the Equations of Life. Harvard university press; 2006.

[7] Santos FC, Pacheco JM, Lenaerts T. Cooperation Prevails When Individuals Adjust Their Social Ties. PLOS Computational Biology. 2006;2(10):1–8. doi: 10.1371/journal.pcbi.0020140 17054392 PMC1617133

[8] Santos FC, Santos MD, Pacheco JM. Social diversity promotes the emergence of cooperation in public goods games. Nature. 2008;454(7201):213–216. doi: 10.1038/nature06940 18615084

[9] Nowak MA. Five rules for the evolution of cooperation. science. 2006;314(5805):1560–1563. doi: 10.1126/science.1133755 17158317 PMC3279745

[10] Sanchez A. Physics of Human Cooperation: Experimental Evidence and Theoretical Models. Journal Of Statistical Mechanics: Theory and Experiment. 2018;2018(2):024001. doi: 10.1088/1742-5468/aaa388

[11] Lloyd WF. WF Lloyd on the Checks to Population. Population and Development Review. 1980;6(3):473–496. doi: 10.2307/1972412

[12] Ohtsuki H, Hauert C, Lieberman E, Nowak MA. A simple rule for the evolution of cooperation on graphs and social networks. Nature. 2006;441(7092):502–505. doi: 10.1038/nature04605 16724065 PMC2430087

[13] Wu B, Zhou D, Fu F, Luo Q, Wang L, Traulsen A. Evolution of cooperation on stochastic dynamical networks. PLOS ONE. 2010;5(6):e11187. doi: 10.1371/journal.pone.0011187 20614025 PMC2894855

[14] Wu B, Zhou D, Wang L. Evolutionary dynamics on stochastic evolving networks for multiple-strategy games. Physical Review E. 2011;84(4):046111. doi: 10.1103/PhysRevE.84.046111 22181231

[15] Li A, Zhou L, Su Q, Cornelius SP, Liu YY, Wang L, et al. Evolution of cooperation on temporal networks. Nature communications. 2020;11(1):1–9. doi: 10.1038/s41467-020-16088-w 32385279 PMC7210286

[16] Allen B, Lippner G, Chen YT, Fotouhi B, Momeni N, Yau ST, et al. Evolutionary dynamics on any population structure. Nature. 2017;544(7649):227–230. doi: 10.1038/nature21723 28355181

[17] Yang G, Csikasz-Nagy A, Waites W, Xiao G, Cavaliere M. Information Cascades and the Collapse of Cooperation. Scientific Reports. 2020;10(1):1–13. doi: 10.1038/s41598-020-64800-z 32409658 PMC7224182

[18] Bikhchandani S, Hirshleifer D, Welch I. Learning from the Behavior of Others: Conformity, Fads, and Informational Cascades. Journal Of Economic Perspectives. 1998;12(3):151–170. doi: 10.1257/jep.12.3.151

[19] Shu F, Liu Y, Liu X, Zhou X. Memory-based conformity enhances cooperation in social dilemmas. Applied Mathematics and Computation. 2019;346:480–490. doi: 10.1016/j.amc.2018.10.055

[20] Liu Q, Zhang X, Li Y. The influence of information cascades on online reading behaviors of free and paid e-books. Library Information Science Research. 2020;42(1):101001. doi: 10.1016/j.lisr.2019.101001

[21] Miles AL, Cavaliere M. Opinion Diversity and the Resilience of Cooperation in Dynamical Networks. Mathematics. 2021;9(15):1801. doi: 10.3390/math9151801

[22] Hirshleifer DA. Informational cascades and social conventions. The new palgrave dictionary of economics and the law. 1988; p. 9705–10.

[23] Loess: Local Polynomial Regression Fitting—RDocumentation;. Available from: https://www.rdocumentation.org/packages/stats/versions/3.6.2/topics/loess.

[24] Leskovec J, Singh A, Kleinberg J. Patterns of Influence in a Recommendation Network. In: Pacific-Asia Conference on Knowledge Discovery and Data Mining. Springer; 2006. p. 380–389.

[25] Maharani W, Gozali AA, et al. Degree Centrality and Eigenvector Centrality in Twitter. In: 2014 8th international conference on telecommunication systems services and applications (TSSA). IEEE; 2014. p. 1–5.

[26] Vosoughi S, Roy D, Aral S. The spread of true and false news online. Science. 2018;359(6380):1146–1151. doi: 10.1126/science.aap9559 29590045

[27] Aral S. The Hype Machine: How Social Media Disrupts Our Elections, Our Economy, and Our Health–and How We Must Adapt. Currency; 2021.

[28] Shirado H. Individual and collective learning in groups facing danger. Scientific Reports. 2022;12(1):6210. doi: 10.1038/s41598-022-10255-3 35418611 PMC9007963

[29] Liu S, Zhao L, Zhang J. Strategy Dynamics with Feedback Control in the Global Climate Dilemma Games. In: 2019 IEEE International Conference on Systems, Man and Cybernetics (SMC). IEEE; 2019. p. 518–522.

[30] Pejo B, Biczok G. Corona Games: Masks, Social Distancing and Mechanism Design. In: Proceedings of the 1st ACM SIGSPATIAL International Workshop on Modeling and Understanding the Spread of COVID-19; 2020. p. 24–31.

